# Superior alternating hemiplegia (Weber's syndrome)- Case report

**DOI:** 10.1016/j.amsu.2022.103674

**Published:** 2022-04-28

**Authors:** Mohamed Sheikh Hassan, Nor Osman Sidow, Bakar Ali Adam, Abdulkamil Abdullahi Adani

**Affiliations:** a Department of Neurology, Mogadishu Somali Turkish Training and Research Hospital, Somalia; b Department of Internal Medicine, Mogadishu Somali Turkish Training and Research Hospital, Somalia

**Keywords:** Hemiplegia, Oculomotor palsy, Midbrain infarction, Weber's syndrome

## Abstract

**Introduction:**

and importance: Weber's syndrome is a rare type of brain stem stroke syndrome that is characterized by ipsilateral oculomotor nerve palsy and contralateral hemiparesis. The most common etiology is a midbrain infarction caused by occlusion of the paramedian branches of the posterior cerebral artery or the perforating branches of the basilar bifurcation. Although there are many multiple brainstem strokes, it is uncommon to see this syndrome.

**Case presentation:**

Here we present a case of a 62-year-old male hypertensive patient who presented with a one-week history of cognitive dysfunction, left hemiparesis, right eye ptosis, and right medial gaze palsy (oculomotor nerve palsy). Diffusion MRI showed milimetric diffusion restriction in the right side of the mesencephalon, consistent with an acute infarct. Based on the clinical and radiological findings, a diagnosis of Weber's syndrome was made. The patient was treated with antiplatelet and Piracetam along with strict blood pressure control. There was a massive improvement in the patient's condition on the follow-up visit three weeks later.

**Clinical discussion:**

Weber's syndrome is a rare brainstem stroke due to midbrain infarction and is characterized by crossing hemiplegia consisting of ipsilateral occulomotor nerve palsy and contralateral limb weakness due to damage to the corticospinal tract. Despite it being a brainstem stroke infarct, it carries a good prognosis if it is early treated along with strict control of the risk factors such as hypertension in this case. Our case had massive clinical improvement within three weeks of medical treatment and risk factor control.

**Conclusion:**

This case highlights the classic rare syndrome of brainstem stroke presenting with crossing hemiparesis due to midbrain infarction. This syndrome has a favorable prognosis.

## Highlights

• A 62-year old male hypertensive patient presents with crossing hemipareis with ipsilateral third nerve palsy sparing the pupil.
• Diffusion MRI revealed right midbrain infarction consistent with Weber's syndrome (rare brain stem syndrome).
• Despite it being a brainstem stroke infarct, our case had massive clinical improvement after medical management and strict risk factor control.

## 1 Introduction

Weber's syndrome was first described in 1863 by the German physician Hermann Weber, after examining a 52-year-old male patient who had combined left 3rd nerve palsy with right hemiplegia due to hemorrhage in the left cerebral peduncle. The clinical finding of Weber's syndrome is crossing hemiplegia, including ipsilateral 3rd nerve palsy and a contralateral limb weakness caused by a lesion in the mid-brain. Since then, certain cases have been reported where a mid-brain lesion can produce a 3rd nerve lesion sparing the pupils [1,7]. A complete oculomotor (3rd) nerve lesion from any cause results in ipsilateral ptosis, pupillary dilatation, loss of pupillary and accommodation reflexes, and lateral deviation of the eye. The pupil may be spared if the oculomotor (3rd) nerve lesion is incomplete. Weber's syndrome occurs when the 3rd nerve palsy is combined with contralateral hemiplegia (crossing hemiplegia) [2,8]. In this case, we present a rare case of brainstem stroke (Weber's Syndrome) in a 62-year old male hypertensive patient who presents with crossing hemiparesis and is medically managed.

## 2 Case report

A 62-year-old male hypertensive patient came to our neurology clinic presenting with a one-week history of cognitive dysfunction, left hemiparesis, right eye ptosis, and lateral eye deviation (right 3rd cranial nerve palsy). On examination, the patient had mild disorientation with a Glasgow coma scale of 13/15 (eye-opening-4, verbal response-5, motor response-4). He had right-sided weakness; muscle strength was 3/5 on the left side. Pupils were equal and reactive to light (pupils were spared despite oculomotor palsy). There was no associated dysphagia, aphasia, dysarthria, or other cranial nerve palsies. The rest of the neurologic examination was normal. He had no other significant past medical or surgical history except uncontrolled hypertension. He had no family history of diabetes; hypertension, coagulopathy, or any other neurological disease. No drug abuse or anticoagulation history was found. Vital signs and laboratory tests, including a complete blood count and a broad biochemistry profile were normal.

**Fig. 1:**
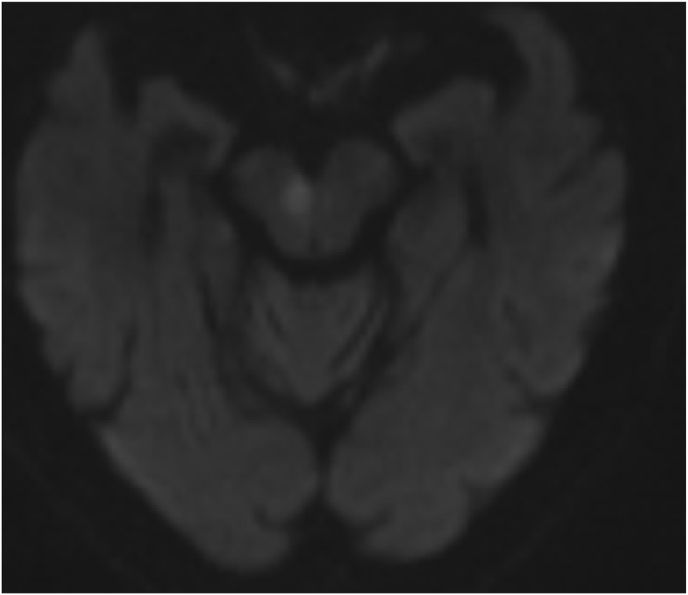
DWI showing hyperintense right midbrain infarction.

**Fig. 2:**
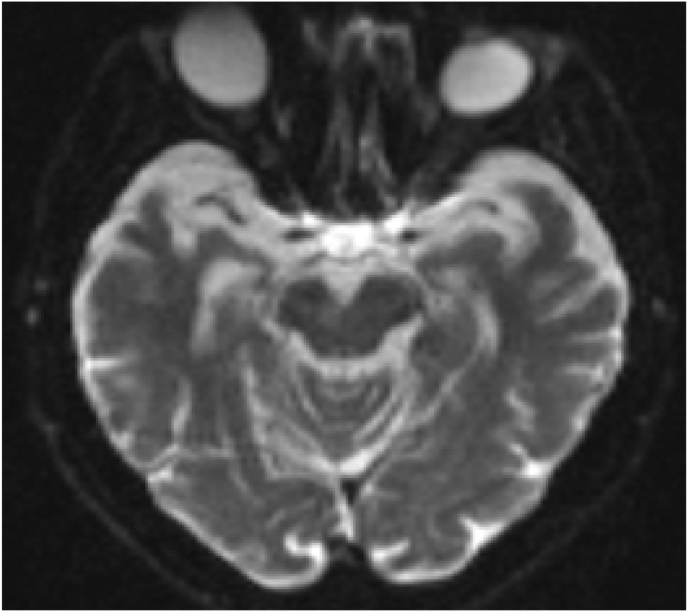
ADC showing hypointense right midbrain infarction.

Diffusion MRI showed milimetric diffusion restriction in the right side of the mid brain consistent with Weber's syndrome (see Fig. 1, Fig. 2). All other tests, including electrocardiogram, echocardiography, as well as carotid and vertebral arteries color Doppler showed normal findings. Based on the clinical and radiological findings, a diagnosis of Weber's syndrome was made.

The patient was treated with antiplatelet (Aspirin 300 mg) and piracetam (Nootropil 800 mg TID) along with strict blood pressure control antihypertensive medications. Three weeks later, the patient clinically improved (cognition improved, hemiparesis diminished, and right 3rd cranial nerve palsy almost disappeared). This case has been reported in line with the SCARE 2020 criteria [11].

## 3 Discussion

A lesion in the ventromedial midbrain causes Weber syndrome. The paramedian mesencephalic branches (basilar), peduncular perforating branches (posterior cerebral artery), the superior cerebellar artery, and the choroidal arteries together supply blood to the midbrain. Weber syndrome is caused by isolated infarctions of the paramedian mesencephalic and peduncular perforating branches. Some of the less common causes include hemorrhage, aneurysms, tumors, and demyelinating diseases [3,9].

The incidence of solitary midbrain infarction causing Weber syndrome is unknown; generally, infarctions in the vertebra-basilar system occur concurrently with infarctions in other locations. Patients with Weber syndrome present with ptosis, diplopia associated with contralateral partial or complete paralysis of the upper limb and lower limb. The presence of crossing sensory or motor impairment, as well as oculomotor nerve palsy, is the hallmark neurological findings of this condition [4]. Our case had sudden onset right ptosis, medial gaze palsy (3rd nerve palsy sparing the pupil), left hemiparesis, and mild cognitive dysfunction. fortunately the patient's condition improved within 3 weeks of medical treatment.

The 3rd nerve nuclei are located in the midbrain and extend approximately 10 mm from rostral to caudal. The Edinger-Westphal nucleus in the upper midbrain supplies fibers to the pupils, and the motor nucleus in the lower midbrain supplies fibers to the extra-ocular muscles. Lesions in the lower midbrain impair extraocular muscles, but pupils are unaffected, whereas lesions in the upper midbrain cause pupillary dilation [5,10]. This explains the pupillary sparing in Weber's syndrome in this patient despite 3rd nerve palsy. The majority of patients with Weber syndrome are neurologically stable.

Early detection, early care, adequate support therapy to prevent complications, and prophylactic precautions to prevent recurrence of similar strokes are the most critical elements influencing the neurological outcome in these individuals [6]. Although our case had a midbrain infarction, he was stable and functional. With correct management, his condition improved within 3 weeks of medical treatment.

## 4 Conclusion

We reported a classic rare case of crossing hemiplegia presenting with left hemiparesis and right oculomotor palsy, sparing the pupil, consistent with Weber's syndrome due to an infarct in the midbrain. Interestingly, our case recovered in 3 weeks, favoring a good prognosis despite in older age. Patients with crossing hemiplegia should be identified and differentiated from other brainstem stroke syndromes.

## Ethical approval

N/A.

## Sources of funding

There is no funding source for this study.

## Author contribution

N.O involved in patient care, collected data, and performed a literature review. M.S.H, wrote the manuscript and also contributed to the patient care. B.A and A.K.A performed literature. All authors reviewed and approved the final version for submission.

## Trial registry number

N/A.

## Guarantor

Mohamed Sheikh Hassan, the corresponding author.

## Consent for publication

Written informed consent was obtained from the patient for publication of this case report and accompanying images. A copy of the written consent is available for review by the Editor-in-Chief of this journal on request.

## Availability of data and materials

N/A.

## Provenance and peer review

Not commissioned, externally peer-reviewed.

## Declaration of competing interest

The authors declare no conflict of interest.
